# Supplementary material for: In-season training responses and perceived wellbeing and recovery status in professional soccer players
Source: PLoS One. 2021 Jul 14;16(7):e0254655. doi: 10.1371/journal.pone.0254655 (PMC8279343; doi:10.1371/journal.pone.0254655)
Supplement: Attachment — Submitted filename: Response to Reviewers.docx [file pone.0254655.s002.docx]

Dear Editor,

Thank you very much for the review of our manuscript entitled: “In-season training responses and perceived wellbeing and recovery status in professional soccer players”. We sincerely appreciate all valuable comments and suggestions, which helped us to improve the quality of the manuscript. Our responses to the Reviewers’ comment are described below in a point-to-point manner. Appropriate changes, suggested by the Reviewers have been introduced to the manuscript (highlighted within the document).

Yours sincerely,

The Authors

**Responses to the reviewer's comments**

Reviewer 3

Please include the limitations on the end of the discussion section. For example: Not including matches information is a relevant limitation in my opinion, once all preparation is for matches.

Thank you. The information was added accordingly (please, see P18 L332). The text now reads: “A second limitation concerns the non-inclusion of match-day information once the balance of training stimulus has a clear focus on the upcoming match.”

More information about the sample must be provided (e.g., VO2max, YOYO performance?). What about the period of the time, number of matches, interval between matches, etc.

Relevant consideration. However, the team studied had important competitive goals. Hence, it is difficult to have those goals compatible with ongoing research. Therefore, the coaching staff required the research team to not influence the training sessions (it was impossible to assess players’ VO2 max). Besides, when asked about the possibility of informing the research team about the results of the assessment tests carried out at the beginning of the pre-season, the club did not authorize our request.

Regarding the data collection period, the number of matches and the interval between matches in which the data was collected, information was added accordingly (please, see P6 L137). The text now reads: “Training load and pre-training wellness indices data for all players were collected over a 16-week in-season period (November-February), without any intervention of the research team regarding training volume and intensity, neither to wellness status perceived by the players (Fig 1). Participants trained on a full-time basis and played competitive fixtures within the Spanish Second Division and Copa del Rey during the 2017-2018 season. Throughout the data-collection period, the team competed in 17 official matches, which often meant that the team played 1 match per week.”.
